# Presynaptic Plasticity as a Hallmark of Rat Stress Susceptibility and Antidepressant Response

**DOI:** 10.1371/journal.pone.0119993

**Published:** 2015-03-05

**Authors:** Jose Luis Nieto-Gonzalez, Mai Marie Holm, Irina Vardya, Trine Christensen, Ove Wiborg, Kimmo Jensen

**Affiliations:** 1 Synaptic Physiology Laboratory, Department of Biomedicine, Building 1160, Aarhus University, DK-8000, Aarhus C, Denmark; 2 Translational Neuropsychiatry Unit, Aarhus University Hospital, DK-8240, Risskov, Denmark; 3 Institute of Neuroscience and Physiology, Sahlgrenska Academy, Gothenburg University, Gothenburg, Sweden; Technion - Israel Institute of Technology, ISRAEL

## Abstract

Two main questions are important for understanding and treating affective disorders: why are certain individuals susceptible or resilient to stress, and what are the features of treatment response and resistance? To address these questions, we used a chronic mild stress (CMS) rat model of depression. When exposed to stress, a fraction of rats develops anhedonic-like behavior, a core symptom of major depression, while another subgroup of rats is resilient to CMS. Furthermore, the anhedonic-like state is reversed in about half the animals in response to chronic escitalopram treatment (responders), while the remaining animals are resistant (non-responder animals). Electrophysiology in hippocampal brain slices was used to identify a synaptic hallmark characterizing these groups of animals. Presynaptic properties were investigated at GABAergic synapses onto single dentate gyrus granule cells. Stress-susceptible rats displayed a reduced probability of GABA release judged by an altered paired-pulse ratio of evoked inhibitory postsynaptic currents (IPSCs) (1.48 ± 0.25) compared with control (0.81 ± 0.05) and stress-resilient rats (0.78 ± 0.03). Spontaneous IPSCs (sIPSCs) occurred less frequently in stress-susceptible rats compared with control and resilient rats. Finally, a subset of stress-susceptible rats responding to selective serotonin reuptake inhibitor (SSRI) treatment showed a normalization of the paired-pulse ratio (0.73 ± 0.06) whereas non-responder rats showed no normalization (1.2 ± 0.2). No changes in the number of parvalbumin-positive interneurons were observed. Thus, we provide evidence for a distinct GABAergic synaptopathy which associates closely with stress-susceptibility and treatment-resistance in an animal model of depression.

## Introduction

Understanding stress susceptibility or resilience of individuals is thought to be important in understanding affective disorders, since this may identify factors that determine individual risks of progressing into a depressive state. Recently, progress has been made in humans and animals identifying some factors that correlate with stress resilience [1–4]. Recent data support the hypothesis that stress-resilience is an active process, and not simply a lack of the changes associated with stress-susceptibility [5]. This type of analysis points to novel pathways, which may be targeted by future antidepressant treatments, in order to shift the balance between resilience and susceptibility [5].

Treatment response or resistance is another important clinical topic that benefits from investigation in animal models [1, 6–8]. Notably, findings suggest similarities between the underlying mechanism of treatment response and stress-resilience based on chromatin changes in the nucleus accumbens [5]. Yet, although some factors have been identified, the underlying function of neuronal microcircuits in stress susceptibility and treatment resistance is poorly understood.

CMS rodent models of depression offer possibilities to study these particular groups of individuals [9]. Indeed, CMS leads typically to anhedonic-like behavior in approximately 50% of the animals, as judged by behavioral tests such as sucrose intake [1, 10, 11]. Furthermore, about half of the stress-susceptible rats respond to antidepressant treatment, such as SSRI administration, leaving a group of treatment-resistant individuals [12, 13]. It is essential to identify cellular and molecular hallmarks that correlate with these behavioral responses [2], since it may open for new mechanistic concepts and therapies.

Inhibitory GABAergic interneurons are important for the neuronal network activity, and are thought to serve as clockwork neurons, as well as neuronal fine tuning devices [14, 15]. Furthermore, several studies link a decreased GABAergic function with the pathophysiology of affective and other neuropsychiatric disorders, both in humans [16, 17] and in animal models [18–20], whereas the use of antidepressants increase the GABAergic tone in the brain [18].

In this context, electrical stimulation of presynaptic fibers is widely used for studies of probability of release, while paired-pulse ratio is used as a relative index of the probability of release from presynaptic terminals [21–24]. According to this, we have previously reported a decreased GABA release probability between control and stress-susceptible rats using electrophysiological recording of dentate gyrus granule cells in brain slices of rat CMS model of depression [20].

Here, we studied GABAergic synapses of dentate gyrus granule cells, and investigated their functional properties in three additional groups (stress-resilient, treatment-responder and non-responder) in ventral hippocampus in a CMS model. Our data show that a GABAergic synaptopathy associates closely with the behavioral state of the animal. We suggest that this synaptopathy has a presynaptic origin and can identify stress-resilient and stress-susceptible individuals, as well as animals not responding to antidepressant treatment.

## Materials and Methods

### Ethics statement

Male Wistar rats (Taconic, Denmark) were kept in a university animal facility with a 12/12-h light/dark cycle with unrestricted access to food and water, except when food or/and water deprivation was applied as a stress parameter, in accordance with Danish and European legislation regarding the welfare of laboratory animals. The standard 12-h light/dark cycle was only changed in course of stress regime. All procedures involving animals were approved by Danish National Committee for Ethics in Animal Experimentation (2002/561–575). Procedures for housing and sacrificing of rats were approved by the Animal Welfare Officer at the Faculty of Health Sciences, Aarhus University. To reduce the number of test animals and due to the low availability number of animals in this model, we pooled some previous data [20] with new data in this manuscript. This applies to both electrophysiology and immunohistochemistry.

### Animals and CMS protocol

Rats weighed ∼200 g when sucrose adaptation was initiated, and approximately 350 g at the start of stress. The animals were adapted to consume a palatable sucrose solution (1.5%). Animals were food and water deprived 14 h before the test, which consisted of 1 h exposure to a bottle with sucrose solution. Based on the baseline sucrose consumption animals were divided into two matched groups (equal mean baseline ± 0.5 g), control and CMS groups, and placed in separate rooms. The CMS group was exposed to 8 weeks of chronic mild stressors. Briefly, this procedure consisted of seven different stressors and lasted from 10 to 14 hours. The mild stressors consisted of interchanging periods of intermittent illumination, stroboscopic light, grouping, food and/or water deprivation, soiled cage, 45° cage tilting and no stressors. The control group was left unchallenged except for 14 hours food and water deprivation before sucrose consumption test. The stress procedure was performed according to a procedure optimized in our laboratory [12]. The operational cut off for the anhedonic-like group is a more than 40% drop in sucrose intake index value, while resilient rats are defined by having a less than 10% drop in sucrose index value. Most of the resilient rats even increase in sucrose intake[25]. The operational cut off for drug response was set to 20% increase in sucrose intake and in response to escitalopram a bimodal segregation into responders and non-responders is reported earlier [12]. After initial three weeks exposure to stress, around 50% of the rats entered an anhedonia-like state observed as a more than 40% reduction in sucrose intake as reported previously [26] and the remaining rats were mainly resilient to stress exposure. The stress-susceptible group was divided into two matched subgroups and subjected to chronic escitalopram or vehicle administration for eight weeks. Stress exposure was continued during the entire period of treatment. An unchallenged control group was administered with vehicle in parallel. Drug or vehicle was administered intraperitoneally every morning. Escitalopram was dissolved in 0.9% saline and was given at dosages of 5 mg/kg/day [12]. According to sucrose intake readouts, treated rats segregated into two groups, responders versus non-responders [12].

### Brain slice preparation and electrophysiology

Rats were deeply anesthetized with isoflurane until the tail-pinch reflex disappeared and were subsequently decapitated. 350 μm thick horizontal brain slices were prepared [27] and transferred to ice-cold artificial cerebrospinal fluid (ACSF) composed of (in mM): 126 NaCl, 2.5 KCl, 2 CaCl_2_, 2 MgCl_2_, 1.25 NaH_2_PO_4_, 26 NaHCO_3_, 10 D-glucose, kynurenic acid (3 mM), ascorbate (0.2 mM), and pyruvate (0.2 mM) (osmolality 305–315 mosmol^.^kg-1), pH 7.4 when bubbled with carbogen (5% CO_2_/95% O_2_). Slices were stored for at least 1 h at room temperature (RT) before recording.

Slices containing the ventral hippocampus were placed in a recording chamber (33 ± 1°C) and perfused with bubbled ACSF at 2–3 ml min^-1^. Granule cells were identified in a custom-built infrared videomicroscope equipped with a 40x water-immersion objective (Olympus, Denmark). Patch-pipettes were pulled from borosilicate glass (outer diameter = 1.5 mm, inner diameter = 0.8 mm from Garner Glass Company, Claremont, CA) on a DMZ Universal Puller (Zeitz Instruments, Munich, Germany, resistance 3–5 MΩ) and filled with solution containing (in mM): 140 CsCl, 2 MgCl_2_, 0.05 EGTA, 10 HEPES, pH 7.2 with CsOH, osmolalility 270–290 mosmol kg^-1^ with sucrose. After obtaining stable gigaseals (>1 GΩ), whole-cell recordings were made from the soma in voltage-clamp mode (-70 mV) using a MultiClamp 700B amplifier. Recordings were discontinued if the series resistance increased by 50% or exceeded 20 MΩ. Currents were low-pass filtered (eight-pole Bessel) at 3 kHz, digitized at 20 kHz, and acquired using a BNC-2,110 DA converter and a PCI-6014 board (National Instruments, Austin, TX) combined with custom-written Lab-WIEW 6.1-based software (EVAN v. 1.4, courtesy of Istvan Mody, University of California, Los Angeles, CA). sIPSCs and mIPSCs were collected from cells in different slices, although wash-in experiments of tetrodotoxin (TTX) were performed to verify the effect of this action potential blocker. sIPSCs and mIPSCs were detected using amplitude detection thresholds of 6–8 pA following visual inspection of all events. To estimate the decay time constant of the IPSCs, a double exponential function was fitted to the average trace, calculating two tau values and their respective amplitudes. The weighted tau was calculated using the equation: τ_w_ = A_fast_ · τ_fast_ + A_slow_ · τ_slow_, where A_fast_ and A_slow_ are the relative amplitudes of each component. This weighted tau we denoted the decay time constant. IPSCs were evoked by paired-pulse stimulation every 10 s using a bipolar matrix microelectrode (FHC Inc., ME, USA) placed in the granule cell layer 200–300 μm from the recorded cell. The stimulation intensity was increased until threshold and kept constant at 20–40% above threshold thereafter. The paired-pulse ratio of average IPSC amplitudes was measured by averaging 10–20 sweeps.

### Immunohistochemistry

After an overdose of sodium pentobarbital (200 mg/ml in 10% ethanol), animals were transcardially perfused with 0.9% physiological saline followed by 4% paraformaldehyde (pH = 7.4). The brains were removed and postfixed overnight in the same solution at 4°C. Brains were transferred to a solution consisting of 30% sucrose (diluted in phosphate buffer, pH = 7.0–7.4) supplemented with 0.005% sodium azide and stored at 4°C until they sank. Horizontal sections were cut through the entire dentate gyrus along the dorso-ventral axis (-9.10 to-3.10 relative to bregma) [28] using a CM3050S cryostat (Leica Microsystems, GmbH, Germany). 40 μm sections were collected in series of every sixth (12–16 sections per subserie) and stored in 25% ethylene glycol and 25% glycerin (in 0.05 M phosphate buffer) at-20°C. Immunohistochemical stainings were carried out to reveal parvalbumin-positive cells. Free-floating brain slices were washed in PBS and endogenous peroxidase activity was blocked with ethanol. Sections were incubated for 1 h at RT in a blocking solution containing 3% normal donkey serum and 0.3% Triton X-100 in PBS. Slices were incubated with primary goat anti-parvalbumin antibody prepared in blocking solution (PVG-214, SWANT, 1:8.000) overnight at 4°C. Secondary antibody (biotinylated donkey antigoat IgG, 1:2.000, Jackson ImmunoResearch) was incubated for 2 h at RT and then with standard avidin-biotinylated peroxidase complex (ABC kit, Vector Laboratories) for 1 h at RT. Peroxidase activity was revealed by 0.02% diaminobenzidine with 0.01% H_2_O_2_ and with 0.04% nickel ammonium sulfate. Finally, the sections were placed on gelatinized glass slides and coverslipped with DPX (Fluka). Images were acquired on a Leica AF6000 LX workstation (10x objective). Different brains were used for electrophysiology and immunohistochemistry.

### Statistical analysis

Electrophysiological values are given as means and standard error of mean (SEM), and n stating the number of neurons. Paired or unpaired t-tests were used to assess statistical differences using a 5% significance level.

## Results

### Paired-pulse facilitation in stress-susceptible, but depression in stress-resilient, rats

**Fig 1:**
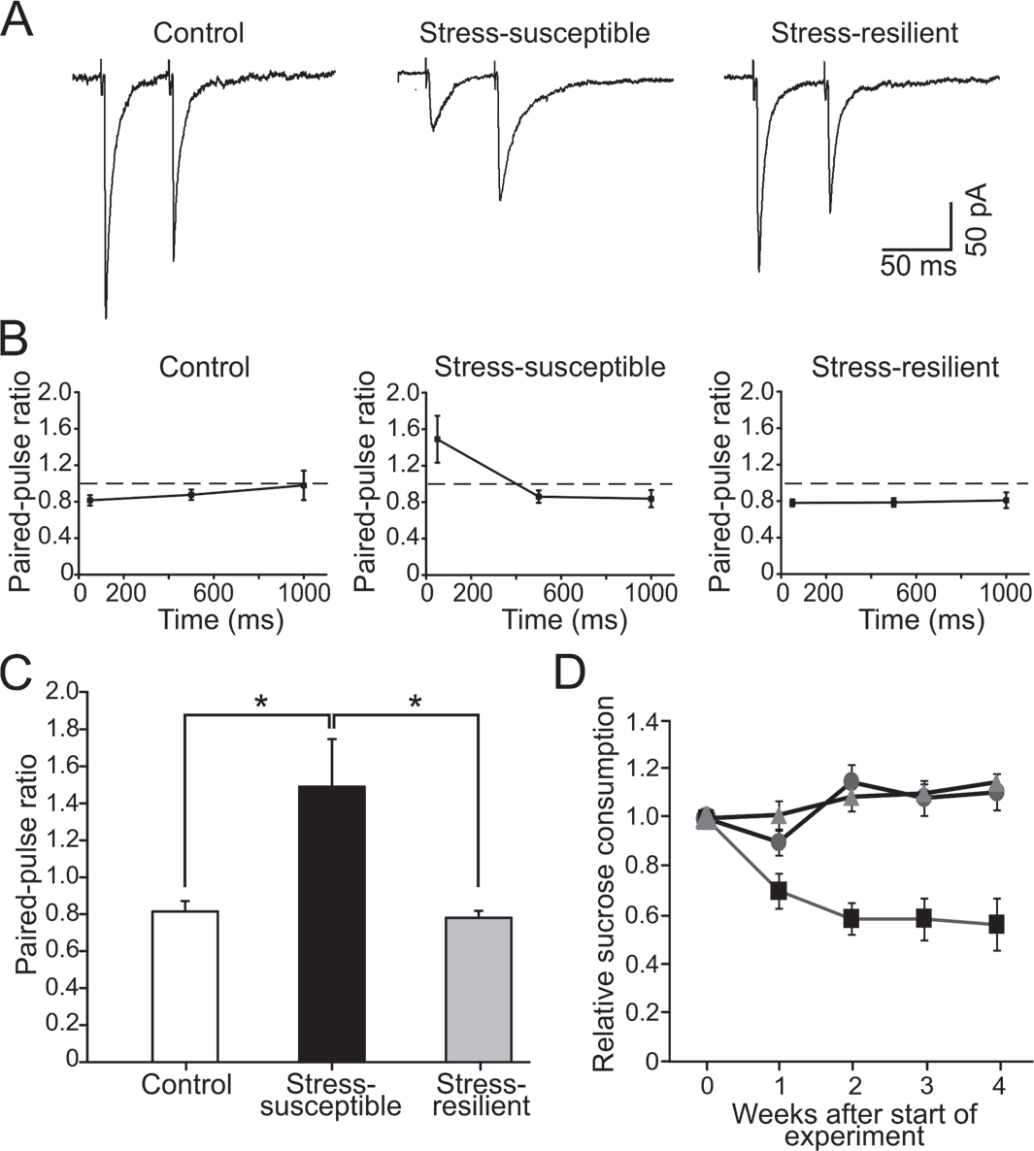
Paired-pulse depression of evoked IPSCs turns into facilitation in stress-susceptible, but not stress-resilient, rat dentate gyrus granule cells. A. Evoked GABA_A_ receptor mediated IPSCs showing paired-pulse behavior in granule cells from unchallenged control, stress-susceptible and stress-resilient rats. Traces are averages of 10–15 sweeps. B. Line plot depicting the paired-pulse ratio at different inter-pulse intervals (50, 500 and 1000 ms) in the three groups. C. Bar graph showing the significant differences (*P* < 0.05) between control (n = 23 cells/6 rats), stress-susceptible (n = 21 cells/6 rats) and stress-resilient (n = 18 cells/6 rats) at a paired-pulse interval of 50 ms. Susceptible rats show paired-pulse facilitation, indicating a reduction in GABA release probability, while resilient rats are similar to control. D. Sucrose consumption for controls (circles), stress-resilients (triangles) and stress-susceptible (squares).

In a search for electrophysiological markers of stress-susceptibility versus resilience, we analyzed the paired-pulse plasticity of electrically evoked IPSCs in dentate gyrus granule cells in CMS rats. Fig. 1A illustrates averaged IPSCs evoked by extracellular stimulation in the granule cell layer in control rats, stress-susceptible and stress-resilient rats. The time course of paired-pulse behavior of IPSCs is shown in Fig. 1B. At a 50 ms interpulse interval, control and resilient rats showed a similar paired-pulse ratio (0.81 ± 0.05, n = 23 versus 0.78 ± 0.03, n = 18). In contrast, the values observed in the stress-susceptible group (1.48 ± 0.25, n = 21) were significantly larger than the two other groups (Fig. 1C, *P* < 0.05). Electrophysiologically, this indicates that the GABA release probability is reduced in stress-susceptible, but not in stress-resilient animals.

### Decreased frequency of sIPSCs in stress-susceptible, but not in stress-resilient, rats

**Fig 2:**
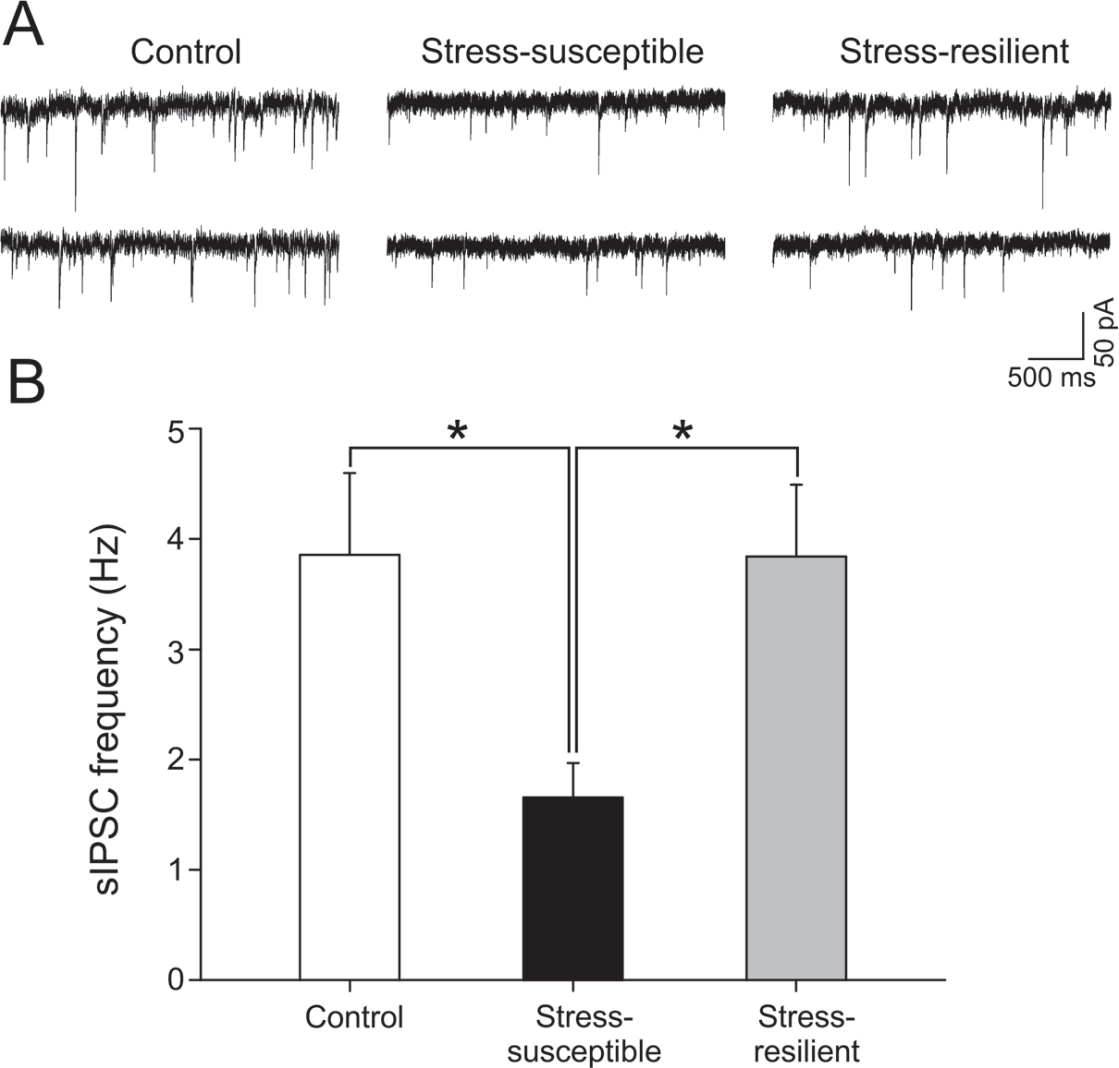
Frequency of spontaneous IPSCs in dentate gyrus granule cells is reduced in stress-susceptible, but not in stress-resilient, rats. A. sIPSC traces recorded in the presence of kynurenic acid (3 mM) to block ionotropic glutamate receptors (V_hold_-70 mV). B. Histogram displaying the significant (*P* < 0.05) reduction in frequency of sIPSCs in stress-susceptible rats (n = 35 cells/10 rats), but not stress-resilient rats (n = 41 cells/9 rats).

To analyze the spontaneous GABAergic activity in the brain slices, we measured the frequency of spontaneous GABA_A_ receptor-mediated IPSCs (sIPSCs). Fig. 2A shows sIPSCs recorded in granule cells from the three groups of rats. Control and resilient rats showed a similar sIPSC frequency (3.86 ± 0.73 Hz, n = 37 and 3.84 ± 0.64 Hz, n = 41, *P* > 0.05, respectively) whereas stress-susceptible rats showed a significantly reduced sIPSCs frequency (1.65 ± 0.3 Hz, n = 35, *P* < 0.05, Fig. 2B).

### No difference in number of parvalbumin-positive interneurons in control, stress-susceptible and stress-resilient rats

**Fig 3:**
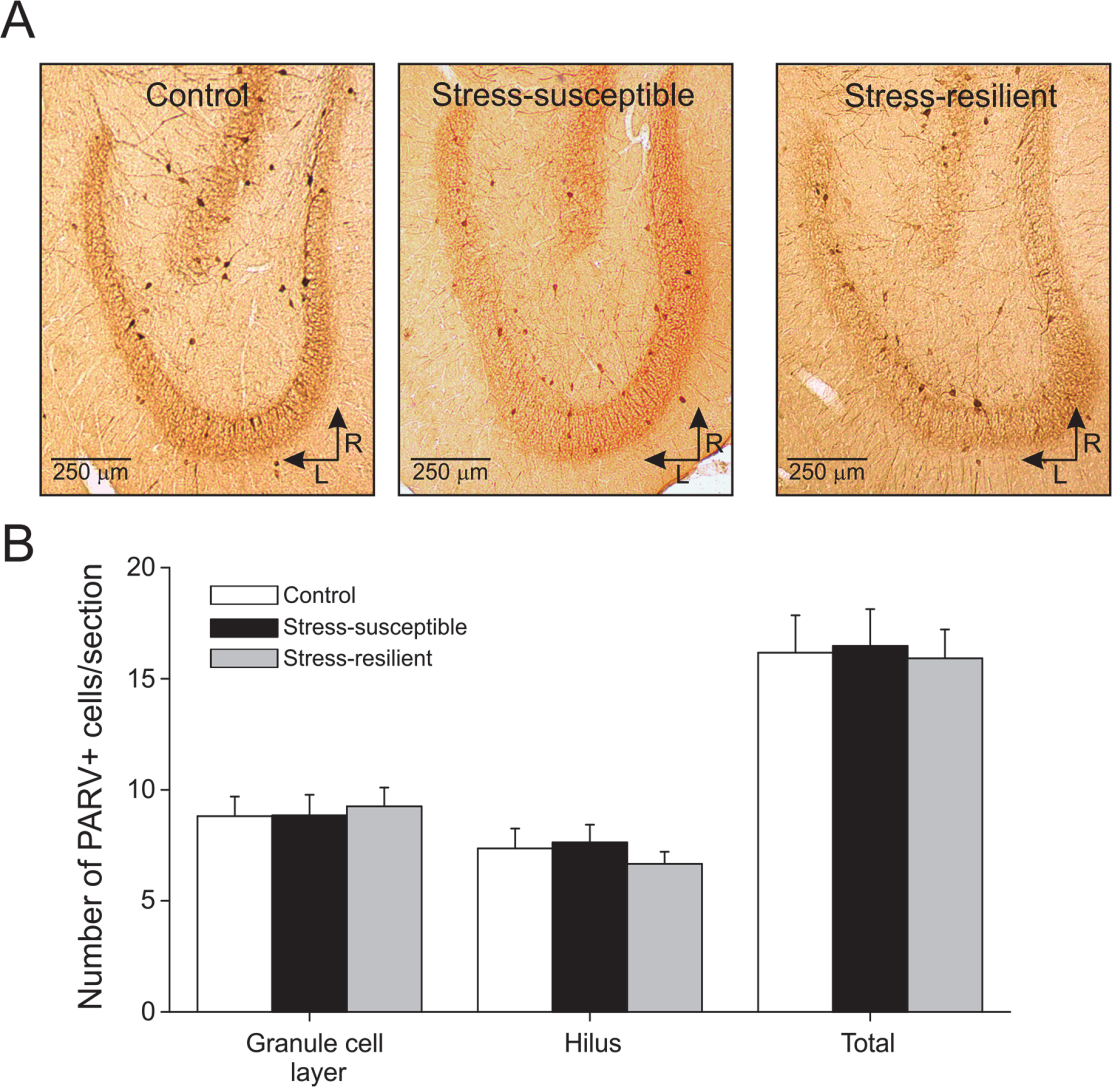
No difference in number of parvalbumin-positive interneurons in dentate gyrus between control, stress-susceptible and stress-resilient rats. A. Immunostainings of parvalbumin-positive interneurons in rat dentate gyrus of control, stress-susceptible and stress-resilient animals (R, rostral; L, lateral). B. Histogram displaying the number of cells per section in the granule cell layer or hilus, and the total number. No significant differences (*P* > 0.05) were found between the groups (PARV+: parvalbumin-positive).

Parvalbumin-containing neurons are considered to be important interneurons responsible for clockwork functions in the dentate gyrus [29], mediate a strong perisomatic input to granule cells [30] and it was previously reported that stress protocols decrease the number of these interneurons in the hippocampus [31, 32]. Therefore, we examined whether CMS changed the number of these interneurons. We counted the number of parvalbumin-positive (PARV+) interneurons in granule cell layer and hilus in control (∼12 sections/rat, n = 10), stress-susceptible (∼12 sections/rat, n = 8) and stress-resilient rats (∼11 sections/rat, n = 10) (Fig. 3A). As shown in Fig. 3B, no significant difference (*P* > 0.05) were found in the number of PARV+ interneuron in granule cell layer or hilus in the 3 groups of rats.

### No difference in miniature inhibitory postsynaptic currents between control, stress-susceptible and stress-resilient rats

**Fig 4:**
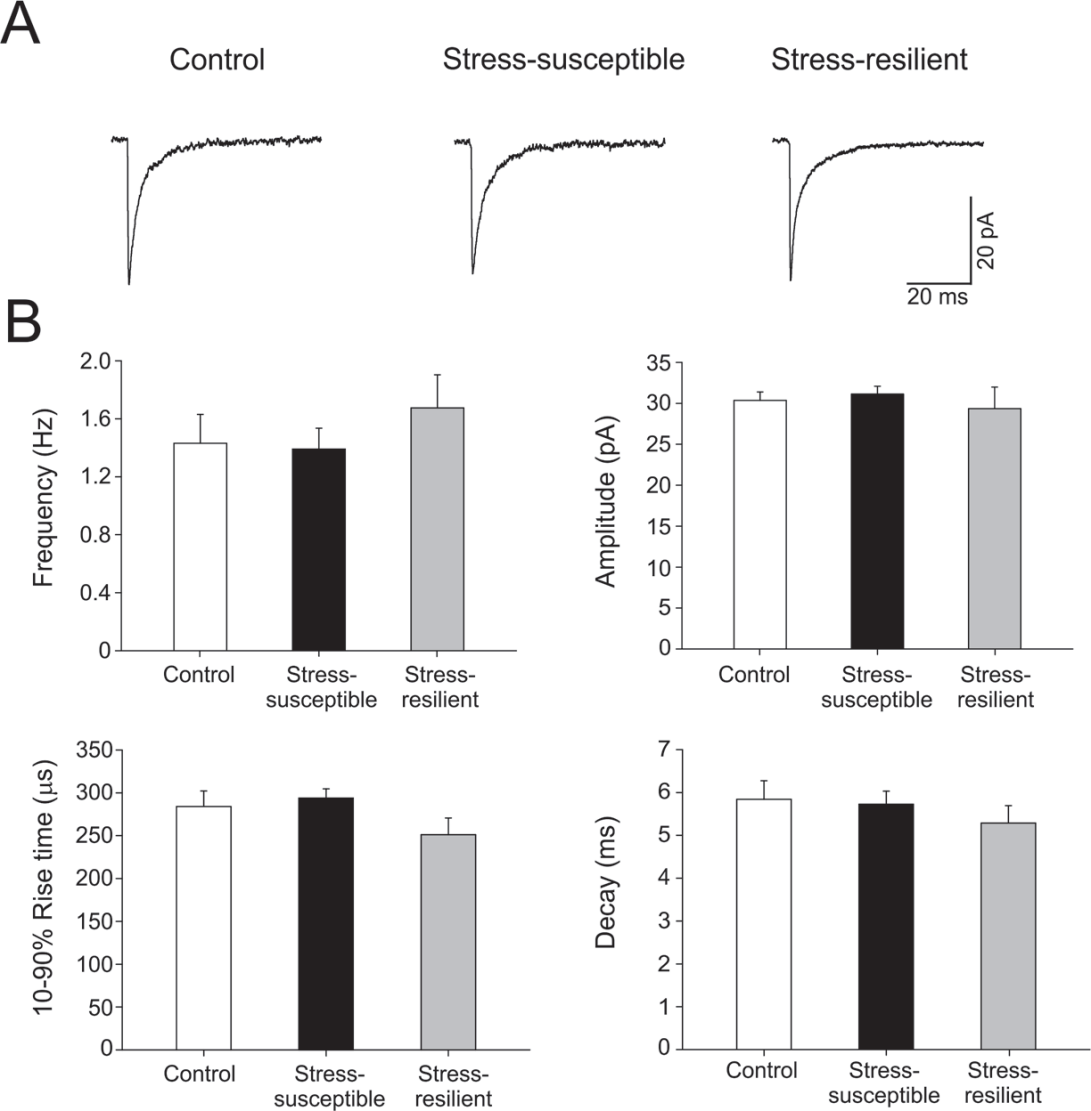
Miniature inhibitory postsynaptic currents (mIPSCs) are similar in control, stress-susceptible and-resilient rats. Miniature IPSCs (mIPSCs) reflect functional properties of single GABAergic synapses under conditions of unitary GABA vesicle release, achieved by blocking action potentials with 1 μM tetrodotoxin (TTX). A. Traces displaying the average mIPSC waveform in unchallenged control, stress-susceptible and stress-resilient animals. B. Average mIPSC parameters including frequency, amplitude, decay time constants and 10–90% rise-time showed no differences (*P* > 0.05) between groups (control n = 13 cells/4 rats; stress-susceptible n = 19 cells/4 rats; resilient n = 11 cells/4 rats), indicating that synaptic GABA_A_ receptor properties in granule cells are unaltered following CMS.

Because no differences were found in the number of PARV+ interneurons, we analyzed miniature inhibitory postsynaptic currents (mIPSCs) to identify a possible dysfunction in the GABAergic terminals. We performed whole-cell recordings of miniature inhibitory postsynaptic currents (mIPSCs) in granule cells in the presence of TTX (1 μM) in order to block action potentials (Fig. 4). To analyze the waveform of mIPSCs, average currents from each cell were constructed (Fig. 4A). As shown in Fig. 4B, control, resilient and stress-susceptible rats displayed similar frequency (1.43 ± 0.2 Hz, n = 19; 1.67 ± 0.22 Hz, n = 11; 1.39 ± 0.14 Hz, n = 26, *P* > 0.05, respectively), amplitude (30.4 ± 1.0 pA, n = 19; 29.4 ± 2.6 pA, n = 11; 31.1 ± 1.0 pA, n = 26, *P* > 0.05, respectively), and rising (284.0 ± 18.1 μs, n = 19; 251.2 ± 19.5 μs, n = 11; 294.0 ± 10.6 μs, n = 26, *P* > 0.05, respectively) and decaying (5.84 ± 0.4 ms, n = 19; 5.28 ± 0.4 ms, n = 11; 5.72 ± 0.3 ms, n = 26, *P* > 0.05, respectively) kinetics indicating unaltered postsynaptic properties. Since the analysis of mIPSCs rules out altered postsynaptic properties, that indicates that the synaptic impairment observe by paired-pulse facilitation in stressed rats is due to a presynaptic alteration in these animals.

### Escitalopram treatment restores GABA release in SSRI responder, but not in SSRI non-responder, rats

**Fig 5:**
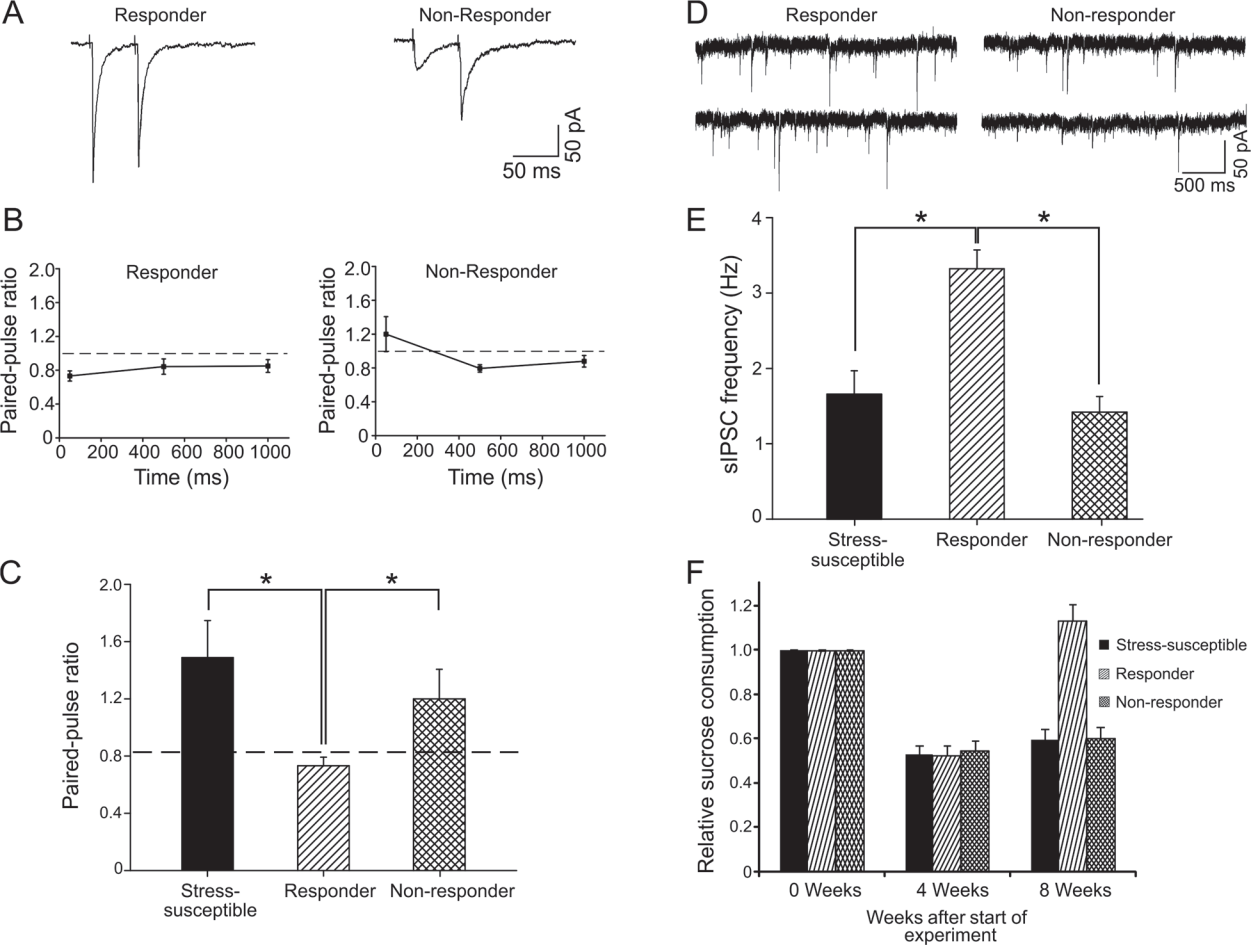
Treatment for 8 weeks with escitalopram restores paired-pulse ratio and sIPSC frequency in a responder subset of stress-susceptible animals. A. Average traces of evoked IPSCs showing paired-pulse depression in responder (n = 9 cells/3 rats) and paired-pulse facilitation in non-responder (n = 18 cells/5 rats) animals. B. Line plot displaying different inter-pulse intervals (50, 500 and 1000 ms) in responder and non-responder animals. Dashed line indicates a paired-pulse ratio of 1. C. Histogram depicting the significant difference (*P* < 0.05) between responder and non-responder in the paired-pulse ratio with a 50 ms inter-stimulus interval. It is noteworthy that non-responder rats showed values not significantly different to stress-susceptible rats. Dashed line indicates the control value. D. sIPSCs recorded from responder (n = 43 cells/11 rats) and non-responder rats (n = 20 cells/6 rats). E. Histogram showing the significant (*P* < 0.05) recovery in responder animals compared with non-responders, which maintained a low sIPSC frequency similar to stress-susceptible rats. F. Relative sucrose consumption in the three groups.

Above, we identified two electrophysiological fingerprints (paired-pulse ratio and IPSC frequency) which could differentiate between stress-susceptible and stress-resilient rats in the CMS model. It has been previously established that escitalopram can reverse the anhedonic-like state in about 50% of stress-susceptible animals (responders), whereas the remaining animals are treatment resistant (non-responders) [10], as judged behaviorally. We tested whether escitalopram could reverse the electrophysiological parameters in brain slices in SSRI responders and non-responders. Eight weeks of treatment with escitalopram led to full recovery of the GABAergic synaptopathy in responder animals and the paired-pulse ratio reached 0.73 ± 0.06 (n = 9), which is not different from controls (*P* > 0.05, Fig. 5A-C). However, in non-responder animals GABA release was not restored, displaying similar values to stress-susceptible animals (1.48 ± 0.25 versus non-responder 1.2 ± 0.2, n = 18, *P* > 0.05) (Fig. 5A-C).

Finally, analyzing sIPSCs we also observed a full recovery of the sIPSC frequency to control levels (control 3.86 ± 0.73 and SSRI responders 3.32 ± 0.24 Hz, n = 43, *P* > 0.05), whilst non-responder rats remained at low sIPSC frequencies not different from stress-susceptible animals receiving no treatment (stress-susceptible 1.65 ± 0.3 Hz and non-responder 1.41 ± 0.2 Hz, n = 20, *P* > 0.05, Fig. 5D,E).

## Discussion

In the rat CMS model of depression, we have identified a distinct presynaptic GABAergic synaptopathy in the dentate gyrus that correlates with the susceptibility to stress leading to an anhedonic-like state. The ventral hippocampus is involved emotional regulation [33] which was our rationale for studying this brain area. Specifically, in stress-sensitive rats the probability of GABA release was lowered, as judged by the observed increase in IPSC paired-pulse facilitation in GABAergic synapses onto granule cells. In contrast, stress-resilient animals showed a normal GABA release probability in conjunction with paired-pulse depression, which was similar to unchallenged controls. The importance of this observation was further substantiated by 8 weeks of escitalopram administration, where stress-susceptible SSRI-responder rats displayed a normalized GABA release, while SSRI non-responder rats retained the GABAergic synaptopathy.

### Mechanisms of stress-susceptibility and resilience in animal models

Several candidate signaling pathways have recently been proposed to be involved in stress resilience [2]. These evidences point to the HPA (hypothalamus-pituitary-adrenal) axis as being involved in the pathogenesis of depression. Corticotropin-releasing-hormone (CRH) is released by the hypothalamus in response to stress induced activation of the HPA-axis, leading to increased release of cortisol from the adrenal glands. In individuals resilient to stress, it is proposed that this response is altered [34]. In humans, there is a tendency to increased levels of cortisol and HPA hyperactivity in depressed patients [35]. Interestingly, our model has indicated that stress-susceptible rats respond with higher corticosterone levels and slower adaptation to stress as compared to stress resilient animals, having lower and faster habituating responses [36]. Glucocorticoid and mineralocorticoid receptors are expressed in the hippocampus and other limbic structures and are affected by the increased levels of corticosterone [37].

There are multiple biological underpinnings for stress resilience. These include adaptive changes in several neural circuits involving numerous neurotransmitters and molecular pathways. In addition to the monoaminergic systems, alterations in neurotransmitter systems may also involve NPY (neuropeptide Y) [2], however, the role of these transmitter systems in the CMS model remains to be elucidated. Finally, increased BDNF (brain-derived neurotrophic factor) was found to associate with stress-induced changes in synapses formed by dopaminergic neurons projecting from the ventral tegmental area to nucleus accumbens, i.e. the mesolimbic reward circuit [4]. Conserved BDNF release in resilient animals was suggested to be caused by an upregulation of presynaptic potassium channels, which was not observed in stress-susceptible animals [4]. It is possible that BDNF-mediated hyperpolarization also underlies the present results, since BDNF is a strong regulator of GABAergic interneuron function in the dentate gyrus [38].

Considering synaptic proteins in our model, excess SNAP (soluble NSF attachment protein), might increase stress vulnerability, since its expression is selectively higher in the stress-susceptible group, compared to both the stress-resilient and the unchallenged control group [1]. In line with this, Wiborg and co-workers investigated the different CMS and control groups by microarray analysis on microdissected tissues from the hippocampal granule cell layer [39]. Interestingly, the synaptic active zone protein RIM1 (Rab3-interacting molecule), which is known to modulate presynaptic plasticity, was also found to be altered, i.e. reduced levels of this protein were shown in anhedonic-vehicle rats and SSRI-non-responders. RIM1 ablation in mice decreases the probability of GABA release, and increases paired-pulse facilitation of glutamate release in CA1 [40], which is in line with a similar finding in the present study of inhibitory synapses in anhedonic-like rats. Finally, although gender-related factors might also be related to depression, being about twice as common in women compared to men [41], in this study only male rats were studied ruling out factors related to the ovarian cycle [42].

### Stress-induced changes of parvalbumin-positive interneurons

Parvalbumin-positive interneurons in the dentate gyrus are believed to be responsible for a large somatic fraction of the action potential driven inhibitory events in granule cells [30], although cholecystokinin-positive interneurons also project onto granule cells [43]. Interestingly, chronic psychosocial stress decreases the number of hippocampal parvalbumin-positive interneurons in the tree shrew [31] and recent findings extended these observations to studies of inhibitory synaptic changes under conditions of high glucocorticoid levels. Zhang et al. found that 30 min of acute restraint stress, or exogenous application of the glucocorticoid receptor agonist dexamethasone, increased sIPSC frequency in CA1 pyramidal cells [32]. Notably, after three weeks of chronic restraint stress exposures, the authors still observed an increased frequency of the sIPSCs, however the number of the parvalbumin-positive interneurons was significantly decreased in all subregions of the hippocampal formation. This reduction in cell number was also reflected in a failure in regulating the rhythmic sIPSCs originating from parvalbumin-positive cells, while cholecystokinin-positive interneurons were left structurally and functionally unaffected [32].

In contrast, we did not find evidence for a major structural impairment of interneuron axonal branching in the dentate gyrus, since the frequency of miniature IPSCs (reflecting the number of GABAergic boutons) was similar, and the number of parvalbumin-positive cell bodies was also unaltered in both stress-susceptible and the stress-resilient group. Generally, mIPSC analysis could support the idea of unchanged bouton number. E.g. if the rate of GABA secretion from individual boutons is unchanged, and bouton number decreases, mIPSC frequency would drop. We think the most feasible explanation for unchanged mIPSC frequency is unchanged bouton number. However, we cannot rule out counterbalancing (and exactly opposite) changes in rate of secretion and bouton number to yield similar mIPSC frequencies, although we think this is unlikely.

The disagreement with Hu et al. (2010), who showed stress-induced impairment in parvalbumin-positive interneurons, is likely to be due to differences in the two stress paradigms used, i.e. chronic restraint stress induces effects diverging from those induced by variable chronic stress paradigms, as rats exposed to chronic restraint stress habituate to the repeated stressor [44]. For the same reason, chronic restraint stress does not induce an anhedonic-like behavior [45]

### Treatment resistance

Treatment resistance has mainly been studied in human patients, but in few animal models. These studies have generally shown that around 35% of patients, depending on cohorts and treatment regimens, do not respond to first-line SSRI treatment [46], and can be defined as treatment resistant. RIM1 expression is increased in CMS animals responding to treatment with escitalopram, whereas expression levels were low in the treatment resistant group. Furthermore, DRP2 (dihydropyrimidinase-related protein 2) dysregulation has been linked to treatment resistance in the CMS model [1]. It will be interesting to investigate the functional relevance of altered expression of distinct presynaptic proteins such as RIM1 for the dentate gyrus synaptopathy in stress-susceptible and treatment resistant CMS rats, and mechanism for their altered expression. Of relevance, RIM1 is receiving increased attention lately due to the generation of knock-outs [47, 48].

### Functional and molecular consequences of the findings

Our electrophysiological findings correlate with the hedonic state of the animal, pointing to similar molecular cascades shared by stress-resilient and treatment responder groups. This has recently been proposed by others [5] and was furthermore indicated in a recent study on our model showing that the two rat groups display strong similarities in protein and RNA expression profiles [1, 39].

Obviously, one could argue that the non-responder group might couple to the absence of the positive changes seen in the responder group with the SSRI administration failing to revert the depressed state in this group of animals. However, the functional findings in our present study, and in particular the paired-pulse data, support the idea that stress-susceptible and treatment resistant groups share underlying molecular mechanisms, which could facilitate development of new lines of antidepressant treatment.

### Pathophysiological consequences

Although many, but not all, studies support the GABA deficit hypothesis in the depression research area [49], we believe our current data add strong arguments for a limbic GABAergic synaptopathy closely being linked to stress-susceptibility, as well as treatment resistance. At the network level, short-term synaptic plasticity is thought to control the likelihood of various activity patterns being transmitted through neuronal networks. I.e. strongly depressing synapses, such as those studied here, will not easily transmit high frequency signals, but act as low-pass filters [50]. The switch from depressing to facilitating synapses could mean that the low-pass filter actually changes to pathological high-pass filtering properties passing through more high-frequency information. Thus, the properties of signal transmission in neural circuitries might be significantly perturbed in stress-susceptible rats, and as well in SSRI treatment resistance.

Whether these distinct GABAergic synaptic alterations are primary causes, or are secondary effects, are yet unknown and the focus of future research. Thus, it will be interesting to examine whether stress-susceptibility and treatment-resistance share similar molecular cascades. Additionally, one could attempt to identify if the stress-resilient animals exhibit endogenous molecular mechanisms similar to what has exogenously been triggered in the treatment responsive group.
